# Supplementary figures and images for: Rescue of impaired blood-brain barrier in tuberous sclerosis complex patient derived neurovascular unit
Source: J Neurodev Disord. 2024 May 23;16:27. doi: 10.1186/s11689-024-09543-y (PMC11112784; doi:10.1186/s11689-024-09543-y)

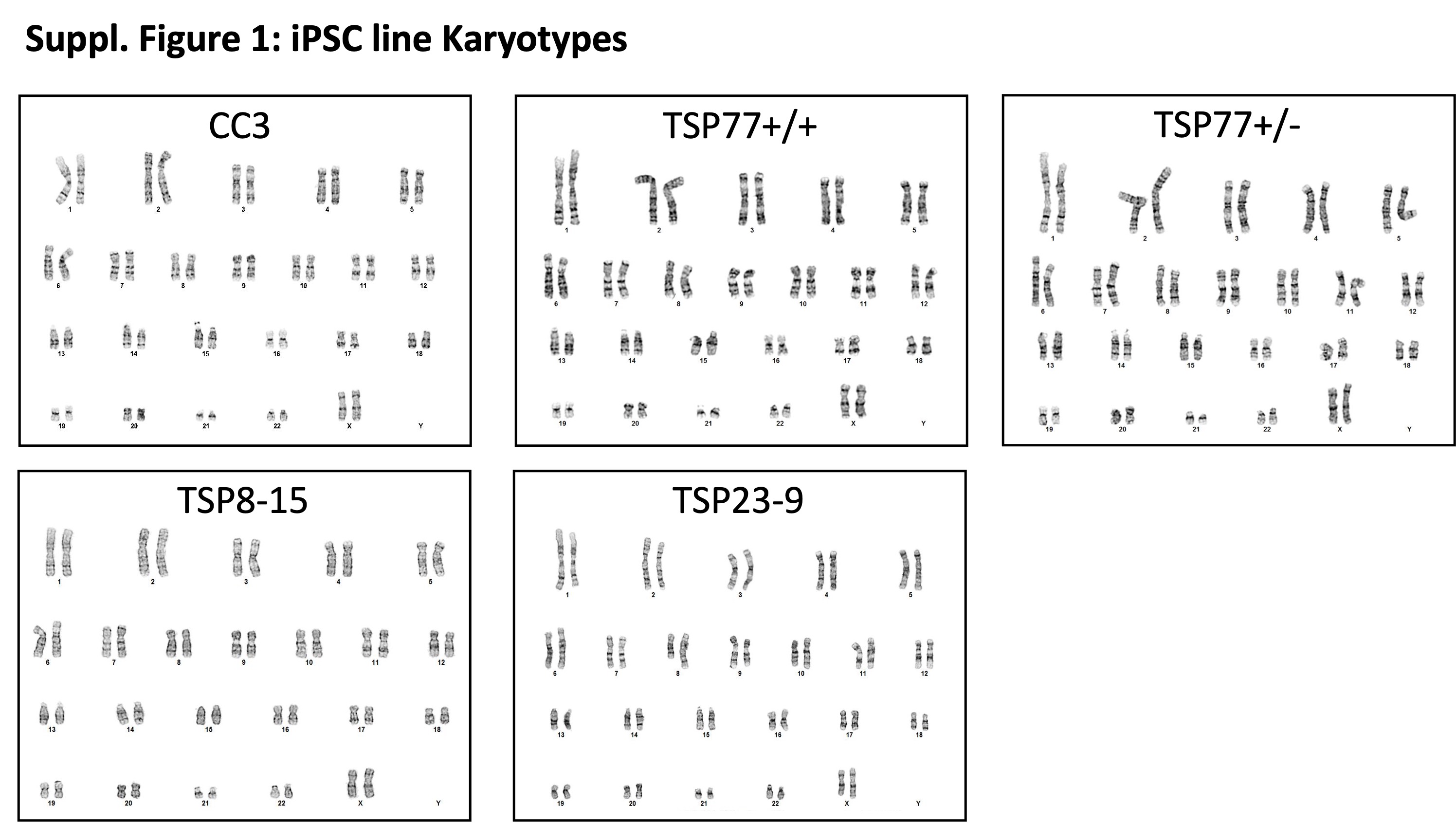

Supplement: Supplementary file 1 — Supplementary Material 1: Supplementary Fig. 1. iPSC Karyotypes. Normal karyotypes of cell lines used. [file 11689_2024_9543_MOESM1_ESM.jpg]

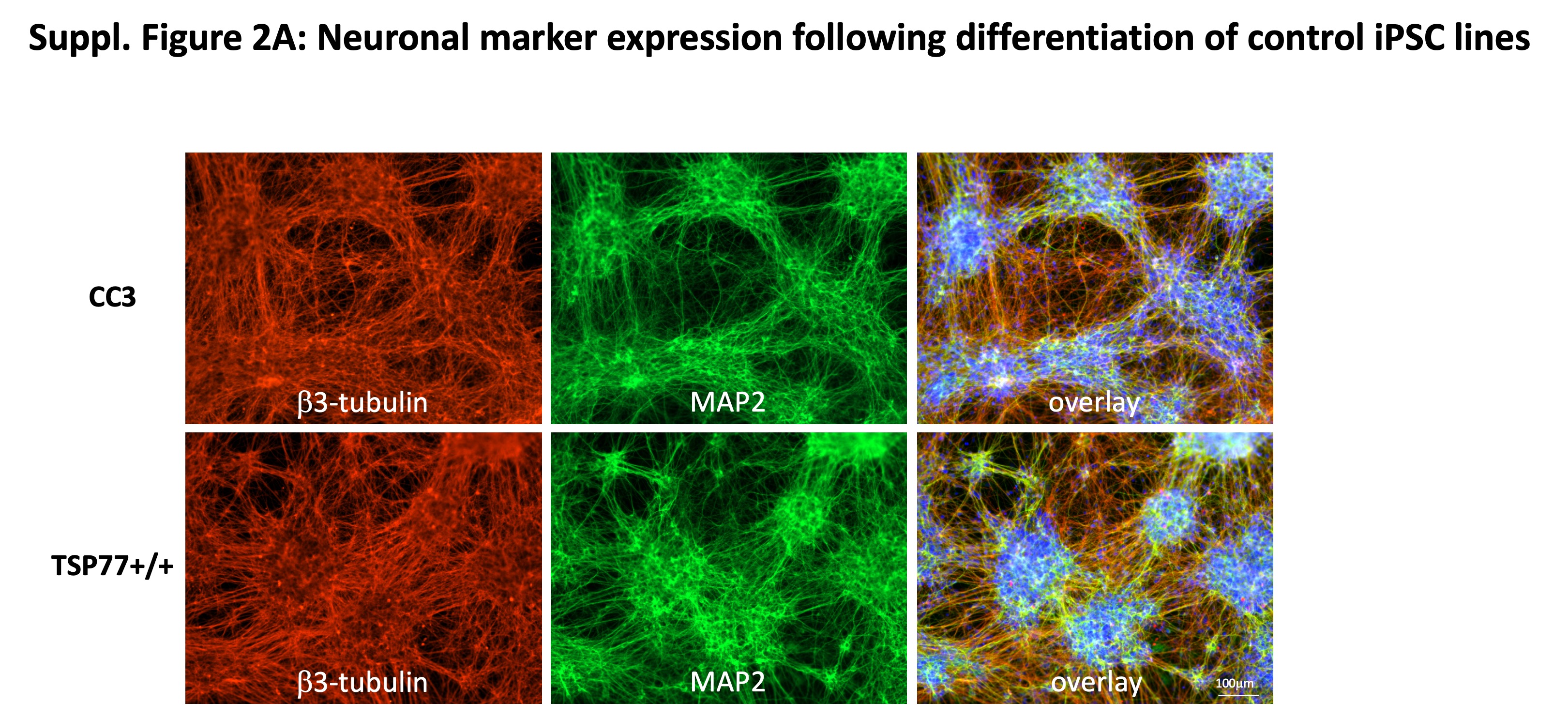

Supplement: Supplementary file 2 — Supplementary Material 2: Supplementary Fig. 2. Cortical neuronal cultures differentiated from control- and TSC- iPSC lines express the neuronal markers β3-tubulin and MAP2. Cortical neuronal cultures differentiated from control iPSC lines (A) and TSC iPSC lines (B) show a dense network of β3-tubulin- and MAP2-positive neurites. The fluorescence signals of the images in Fig. 2 were enhanced for optimal visualization of the markers; therefore, comparisons of expression levels between the different iPSC lines are not possible from this Figure. We have not observed any obvious and consistent genotype-dependent difference in the level or distribution of β3-tubulin or MAP2 expression. TSP77+/+ and TSP77+/- are isogenic TSC wild type and TSC2 heterozygous mutated iPSC lines, respectively. The neuronal cultures shown here were differentiated between 67-104 days before seeding into the NVUs. Scale bar is 100 µm. [file 11689_2024_9543_MOESM2_ESM.zip › SuppleFigure 2A.jpg]

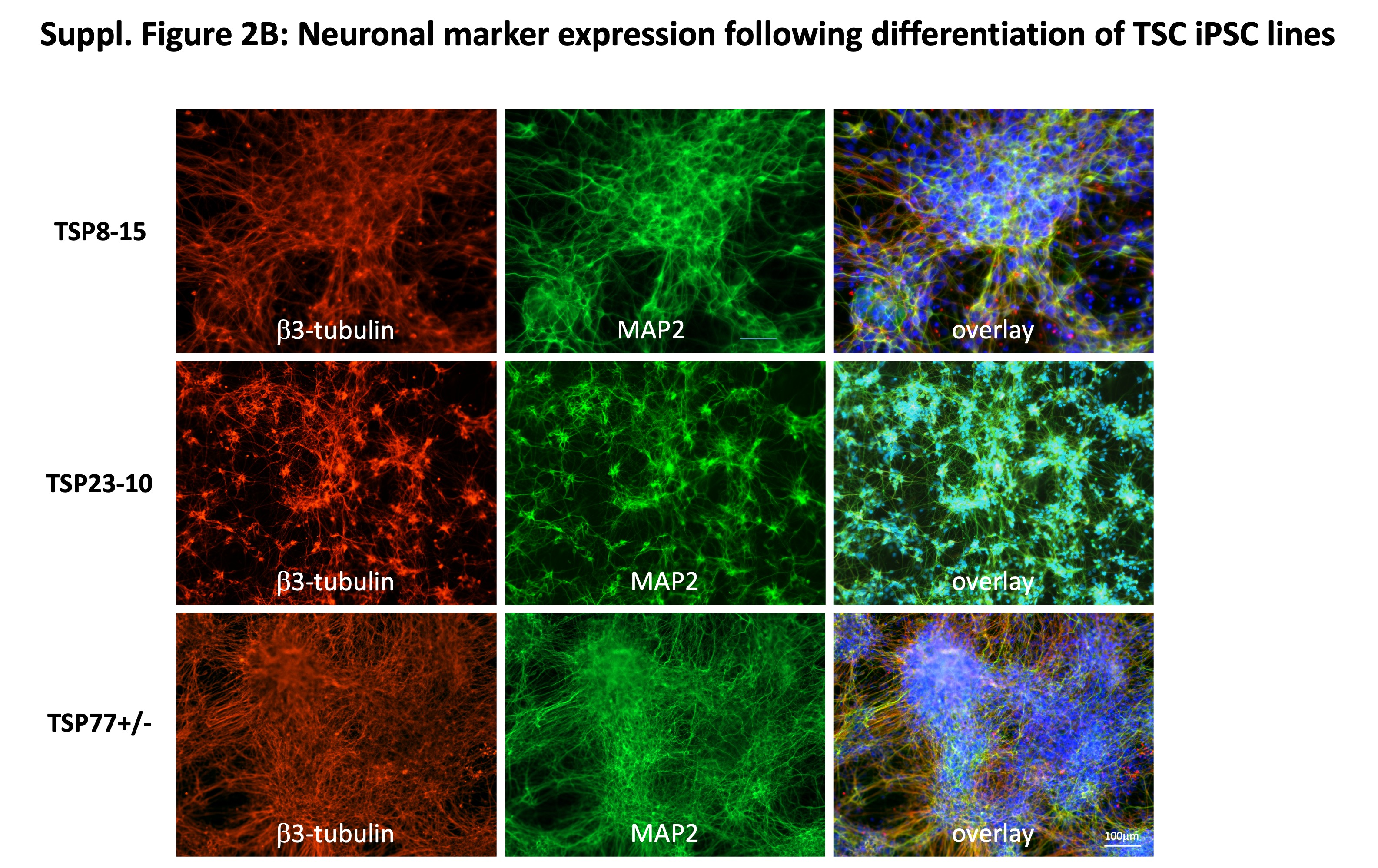

Supplement: Supplementary file 2 — Supplementary Material 2: Supplementary Fig. 2. Cortical neuronal cultures differentiated from control- and TSC- iPSC lines express the neuronal markers β3-tubulin and MAP2. Cortical neuronal cultures differentiated from control iPSC lines (A) and TSC iPSC lines (B) show a dense network of β3-tubulin- and MAP2-positive neurites. The fluorescence signals of the images in Fig. 2 were enhanced for optimal visualization of the markers; therefore, comparisons of expression levels between the different iPSC lines are not possible from this Figure. We have not observed any obvious and consistent genotype-dependent difference in the level or distribution of β3-tubulin or MAP2 expression. TSP77+/+ and TSP77+/- are isogenic TSC wild type and TSC2 heterozygous mutated iPSC lines, respectively. The neuronal cultures shown here were differentiated between 67-104 days before seeding into the NVUs. Scale bar is 100 µm. [file 11689_2024_9543_MOESM2_ESM.zip › SuppleFigure 2B.jpg]

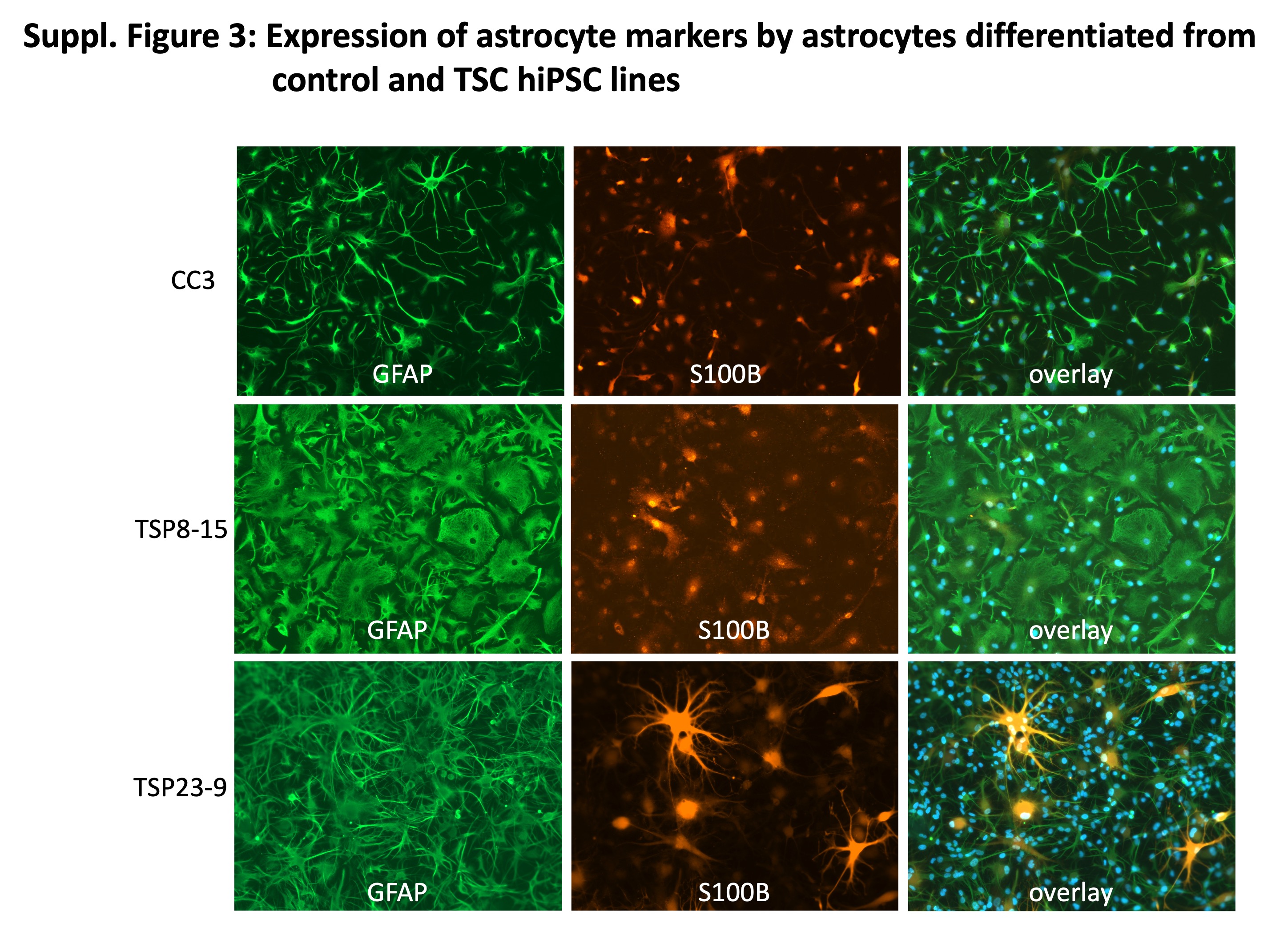

Supplement: Supplementary file 3 — Supplementary Material 3: Supplementary Fig. 3. Astrocyte cultures differentiated from control and TSC-iPSC lines express the astrocytic markers GFAP and S100B. Astrocyte cultures differentiated from control iPSC lines and TSC iPSC lines show expression of GFAP and S100B. The expression levels of both GFAP and S100B varies from cell to cell within the same culture. A few cells express detectable levels of GFAP, but no S100B, some cells express S100B, but no GFAP and the majority of cells express both markers. Generally, the expression of S100B in control and TSC astrocytes co-expressing GFAP is lower than in cells expressing S100B only. The signals of the images were enhanced for optimal visualization of the markers and cell morphology; therefore, comparisons of expression levels between the lines are not possible from this Figure. However, we have not observed any obvious and consistent differences in GFAP or S100B expression between genotypes. TSP77+/+ and TSP77+/- are isogenic TSC2 wild type and TSC2 heterozygous mutated iPSC lines, respectively. The astrocyte cultures shown here were differentiated for >200 days. Scale bar is 100 µm. [file 11689_2024_9543_MOESM3_ESM.jpg]

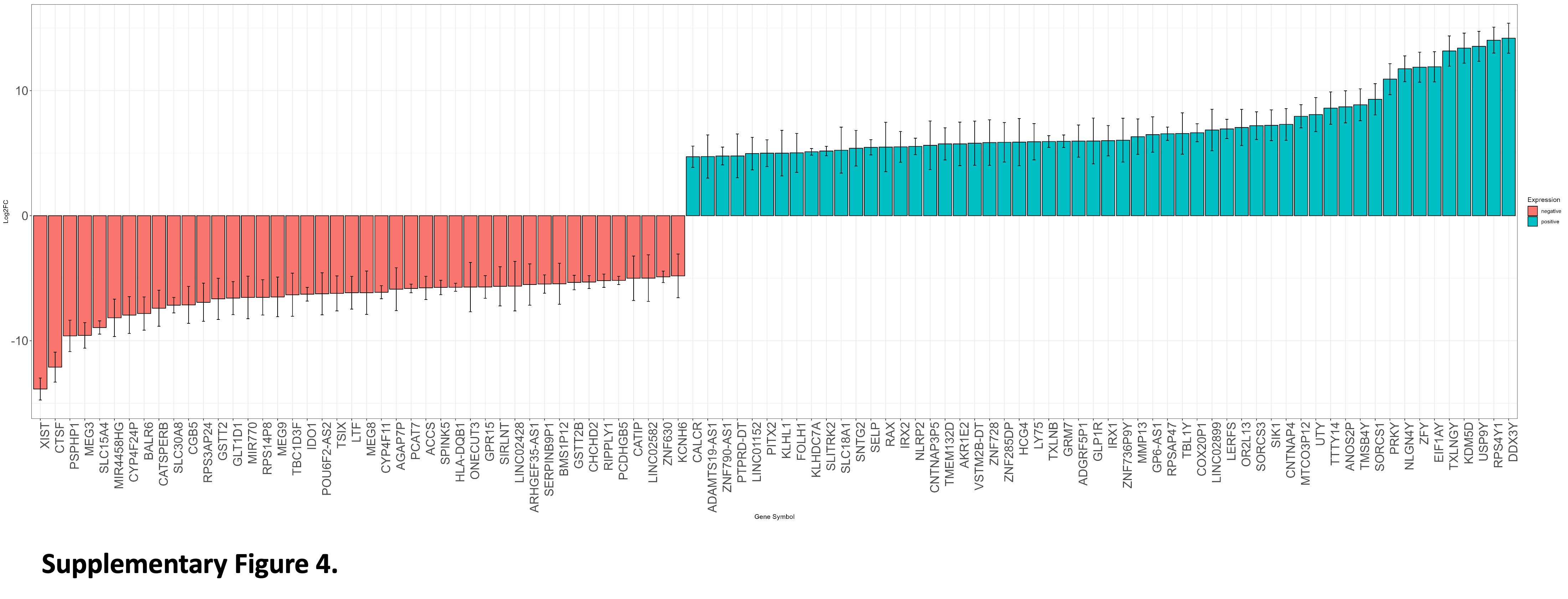

Supplement: Supplementary file 4 — Supplementary Material 4: Supplementary Fig. 4. Genes expressed differentially in control (CC3) and TSPC-patient (TSP8-15) derived NVU neural chambers. The top 100 genes sorted by fold-difference with increased (blue-green) or decreased (orange) expression levels in the neural chamber of TSC2 mutant (TSP-15) compared to control (CC3) are shown. [file 11689_2024_9543_MOESM4_ESM.jpg]

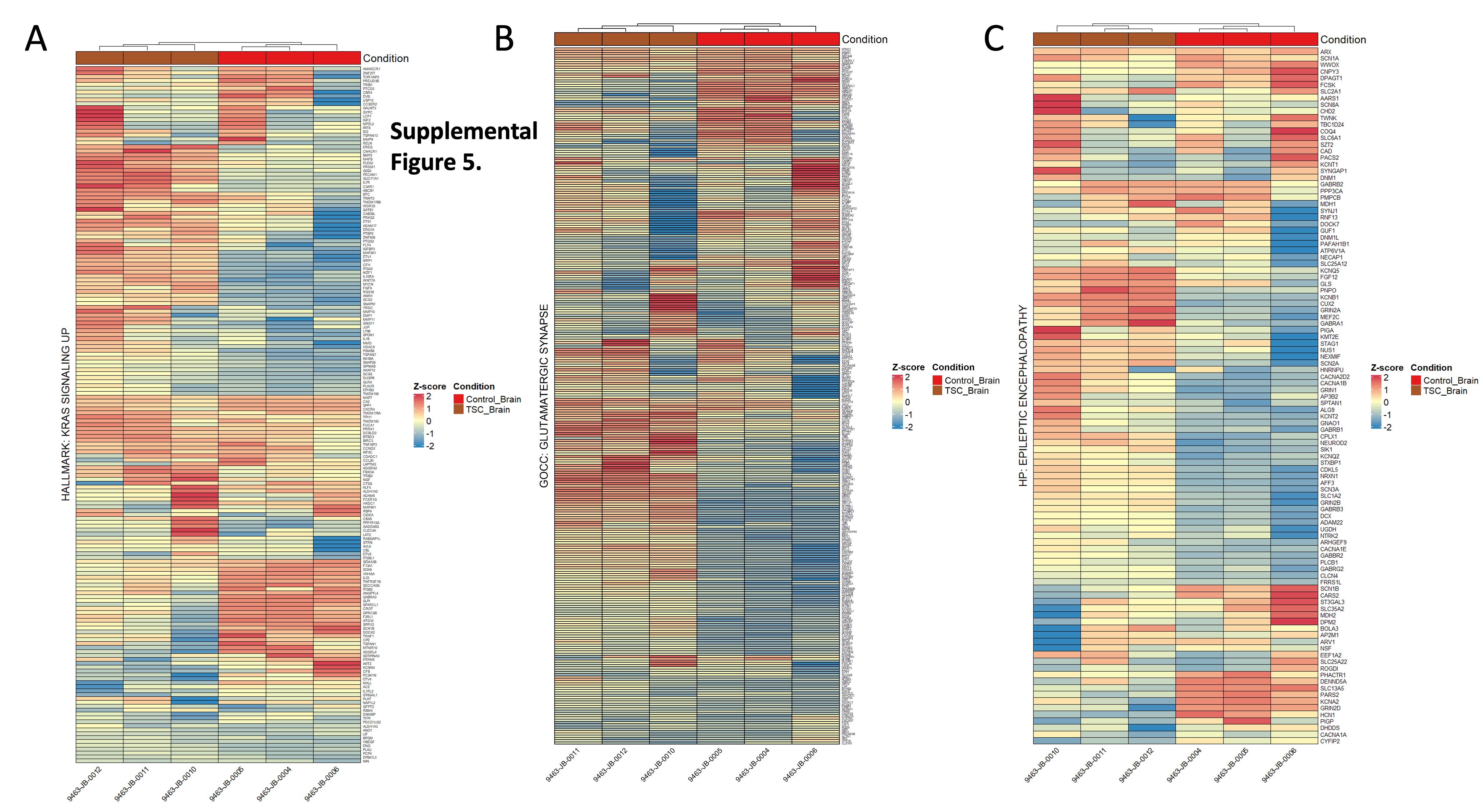

Supplement: Supplementary file 5 — Supplementary Material 5: Supplementary Fig. 5. Gene set analysis of three gene pathways potentially relevant to TSC pathogenesis. We provide gene set analysis for three pathways that might be relevant to TSC CNS pathogenesis: K Ras signaling (A, NES = 1.69, setSize 178), Glutamatergic Synapse (B, NES = 2.32, setSize: 306) and Epileptic Encephalopathy (C, NES = 2.26, setSize 105). [file 11689_2024_9543_MOESM5_ESM.jpg]
